# The Cost of Small-Pox

**Published:** 1903-10-03

**Authors:** 


### The Hospital.

A Journal of

The Medical Sciences and Hospital Administration.

Vol. XXXV.—No. 888. OCTOBER 3, 1903.

### THE COST OF SMALL-POX.

We return this week to the condensed history of recent small-pox epidemics in London which has been compiled by Mrs. Garrett-Anderson from the reports of the Metropolitan Asylums Board, and proceed to consider the extent to which the present method—that of dealing with such epidemics when they come instead of preventing them from coming—affects the pockets of the ratepayers concerned. Mrs. Garrett-Anderson points out that it is impossible to separate expenditure incurred in anticipation of an epidemic, as in the provision of small-pox hospitals, from that which is incurred in controlling the epidemic when it occurs, except by a comparison of the expenditure of ordinary years with that of years of small-pox prevalence. The capital sums borrowed in order to provide accommodation and means of transport for London small-pox patients, on the present method, and down to September 1902, amount to £902,757, of which all but £11,000 has been borrowed since 1883. Of the total debt, £724,698 is still owing, and the balance is being paid by a sinking fund which, together with the interest, calls for about $5\frac{1}{2}$ per cent. on the loan as an annual charge upon the rates. By comparing the current expenditure of epidemic years with that of non-epidemic years shortly before or after, we may arrive at some estimate of the cost of an outbreak; but, in making such a comparison, it is necessary to remember that at any moment an epidemic may commence, and that accommodation must be provided in readiness to cope with it. An adequate staff can neither be improvised, nor transferred with safety from the other hospitals of the Asylums Board, and the staff required for a small-pox hospital is very large. A hospital for 500 small-pox patients requires a staff of 325 persons, including ambulance officials, porters, wharf attendants, clerks, servants, nurses, and doctors. Notwithstanding, however, that what may be called non-epidemic years are heavily taxed for small-pox purposes, it appears, on contrasting the epidemic years 1883–4–5 with the non-epidemic years 1880–1–2, that the former show an increase of £148,860 on the capital account, and of £483,560 on the current account. The epidemic years 1901–2, compared with the non-epidemic years 1899–1900, show an increase of £877,810 on the capital, and of £290,477 on the current account. The extra capital sum due solely to small-pox in the years 1901–2 was £658,260, of which the repayment is spread over several years.

The main element of variety in the maintenance-rate of the Asylums Board for any given year may easily be shown to be furnished by the varying number of small-pox patients. The maintenance-rate in the three epidemic years 1883–4–5 amounted in the aggregate to $3\frac{3}{8}$d. in the pound, while in the three following non-epidemic years it was only $1\frac{3}{4}$d. in the pound. The effect of the last epidemic on the rates was less conspicuous than that of its precursor, but only because the annual charge for old debts has become so great that a moderate addition to the maintenance is hardly noticed. When the hospitals have been provided and supported the cost to the public of epidemic small-pox is but at its commencement. Mrs. Garrett Anderson estimates the loss to the community entailed by the deaths from small-pox of 1,634 people in 1901–2 at £225,800; and she calls attention to the effect of an epidemic in diminishing business or in diverting it into strange channels. This influence is difficult to trace in the metropolis, on account of its mere magnitude, but in country places it is always noteworthy, and is often not recovered from for many years. We know of an instance in the case of a flourishing Western town, an important seat of the clothing trade, where there was no epidemic, but only a few cases and a widely-spread alarm. The shopping of ladies resident within a three or four-mile circle was almost entirely withdrawn, and diverted to a neighbouring city eight or nine miles away; and we were assured, three years later, by a leading draper in the town in which the panic arose, that the local shops had never recovered the effect. The habit of going farther afield had been started, and it continued to prevail. Mrs. Garrett Anderson calls attention also to the cost of panic vaccination, and takes her examples from Islington and from Battersea. In 1899 the public vaccinators of Islington were paid £776, and the vaccination officers £351. In 1902 the public vaccinators were paid £5,204, and the vaccination officers £557. In Battersea, where there were 220 cases and 29 deaths, the total cost of the epidemic was estimated at £15,693 by the Medical Officer of Health, who put down £2,526 for vaccination. The whole cost to Battersea amounted to a little more than £71 for every case of small-pox within the borough, and yet, a short time after the close of the epidemic, the walls in the locality were placarded by handbills purporting to instruct the residents how they might avoid or escape vaccination.

It would appear from the preceding figures that the financial burden of small-pox in London is but little less than a million sterling annually, with occasional aggravations of its weight; and Mrs. Garrett Anderson very pertinently asks why, if we can afford the money, it should not be devoted to some better purpose than that of defraying the cost of epidemics which need not be permitted to occur. She suggests hospitals and sanatoria as objects upon which the million might be better spent. Her figures seem to show that the *Times* paper materially understated the facts when it declared the existence of the Anti-Vaccination League to be at least equivalent to an additional penny upon the income tax, and to show also that the organisation in question is a very definite and heavy burden upon the industry of the country. It is a burden, moreover, from which our great industrial rival, Germany, takes care to keep herself free.

